# A large-scale analysis of sex differences in facial expressions

**DOI:** 10.1371/journal.pone.0173942

**Published:** 2017-04-19

**Authors:** Daniel McDuff, Evan Kodra, Rana el Kaliouby, Marianne LaFrance

**Affiliations:** 1 Microsoft Research, Redmond, United States of America; 2 RisQ, Inc. Cambridge, United States of America; 3 Affectiva, Inc., Waltham, United States of America; 4 Yale University, New Haven, United States of America; Universitatsklinikum Tubingen, GERMANY

## Abstract

There exists a stereotype that women are more expressive than men; however, research has almost exclusively focused on a single facial behavior, smiling. A large-scale study examines whether women are consistently more expressive than men or whether the effects are dependent on the emotion expressed. Studies of gender differences in expressivity have been somewhat restricted to data collected in lab settings or which required labor-intensive manual coding. In the present study, we analyze gender differences in facial behaviors as over 2,000 viewers watch a set of video advertisements in their home environments. The facial responses were recorded using participants’ own webcams. Using a new automated facial coding technology we coded facial activity. We find that women are not universally more expressive across all facial actions. Nor are they more expressive in all positive valence actions and less expressive in all negative valence actions. It appears that generally women express actions more frequently than men, and in particular express more positive valence actions. However, expressiveness is not greater in women for all negative valence actions and is dependent on the discrete emotional state.

## Introduction

While the stereotype that women are more expressive than men is generally supported by the literature [1–6], the reality is more nuanced. With respect to smiling, there is strong evidence that women smile more than men. Not only have women been found to smile more in a large meta-analysis [7] but a number of investigators have found that women exaggerate facial displays of positive emotion [8], with smiling being the most common indicator. With respect to most facial actions however, it is less clear whether women will display more activity than men.

On the one hand, reviews of the literature conclude that women are generally more expressive than men [1, 2, 5, 6] as well as being generally better senders of nonverbal information than are men [6, 9]. Thus we might expect women to express all facial actions more frequently.

On the other hand, women might actually inhibit the expression of certain negative valence actions relative to men. For instance, evidence suggests that women are less likely to display and more likely to suppress overt expressions of anger [10, 11]. This difference has been attributed to perceptions of dominance and affiliation [12]. Overall, from prior research one might expect women to express some facial actions more frequently but others less frequently than men.

Studies of facial muscle activity have found that women show stronger zygomaticus (smiling) and frontalis (brow raising) than men [13, 14]. However, the results for corrugator (brow furrowing) are less consistent. It was found that women showed greater activity when shown videos of snakes [15] and when imagining emotional situations [13]. But in another study women did not consistently show increased corrugator activity in reaction to images of facial expressions than men did [2].

Fewer results are available for lip corner depressor activity. Soussignan et al. [14] observed more depressor activity from women when exposed to averted fear faces than men. However, there is little other empirical observational evidence to support conclusions for this and many other facial actions. Furthermore, there are few studies that examine gender differences in these facial actions in natural contexts and when people are exposed to everyday (non-extreme) emotional stimuli. Finally, the size of these studies has typically been limited to between 10 and 100 participants due to the poor scalability of traditional research methods. With the large interpersonal variability that exists within facial behaviors, effects may have been missed within such small populations.

However, whilst analyzing facial actions alone is helpful, and the facial action coding system (FACS) [16] is a very useful objective taxonomy of facial behavior, it is nevertheless useful to consider how these behaviors are related to underlying emotions. While researchers continue to debate the nature of the link between discrete felt emotion and observed facial display [17–23], there is strong evidence of links between facial behaviors and emotional valence [24]. Kassam [25] performed a systematic study of FACS actions units (AUs) and found strong associations between emotional valence for a subset of AUs. Studies of facial electromyography (EMG) have led to similar findings [26, 27].

Whatever one’s position on the link between facial actions and expressions, there is consensus that facial expressions are often modified by the operation of learned display rules which are the norms that regulate how, when, and where emotions can or should be expressed [28]. The result is that observed differences in facial expressions between individuals or groups in response to the same eliciting event may be due in part to the application of social norms that inhibit or amplify basic facial expressions.

Based on the previous literature we hypothesize that: 1) women will generally express facial action units more frequently than men, 2) differences between men and women will be greatest for actions associated with positive valence, 3) men will express actions associated with states of anger more than women. In order to test our hypotheses about gender differences we required a large dataset of coded facial behavior from men and women. Furthermore, we wanted to collect data in everyday settings, something that is rarely done in studies of nonverbal behavior. To collect these data we took advantage of two methodological innovations. We use an Internet-based method to collect facial expression data in naturalistic settings on a very large scale and apply automated facial action coding to quantify the collected facial reactions.

## Method

### Materials

Two hundred and thirty video advertisements aired over the past 10 years were used as stimuli (mean duration: 27.3 seconds; sd: 8.65 seconds). These ads were selected to represent typical online content and covered a range of product categories (confectionery, instant foods, pet foods and cosmetics). The list of confectionery ads was made up of 120 commercials from 31 brands (e.g., Mars, Snickers, Twix, Kinder). Twenty ads representing seven brands were taken from the instant foods category (e.g., Uncle Ben’s, Dolmio, Swartz). The pet foods category consisted of 68 ads from 12 brands (e.g., Pedigree, Cesar, Frolic, Kitekat). The cosmetics category consisted of eight ads from seven brands (e.g., Pantene, Triple Velvet, Old Spice). The remaining 14 ads were taken from beverage (5), automotive (3) and I.T./services (6) ads from 11 brands.

In order to test whether gender effects were consistent across different cultures we collected data from five countries (France, Germany, UK, US, China). Viewers from each country only watched ads from their country and in the official language of their country. Of the 230 ads: 40 ads were from France, 70 ads were from Germany, 40 ads were from the UK, 60 ads were from the US and 20 ads were from China. The ads from different categories were more or less evenly distributed across the different countries.

### Participants

Participants were recruited from an online market research panel between September 2012 and December 2012. In order to take part, participants were required to be at least 16 years of age and have a functioning webcam attached to their computer. At least 70% of the viewers who watched each ad were a user of the product category being advertised which was assessed at the start of the survey. These were the only inclusion/exclusion criteria applied.

The samples were recruited so as to have approximately equal numbers of men and women and represent a range of ages. The participants recruited from each country watched advertisements from that country. The respondents were compensated with approximately $8.10 for participating (depending on the country’s currency). Participants completed the survey in one sitting that took a median time of 26 minutes. In total, 2,106 (ages 16 to 82) people watched 10 ads and completed the survey. We do not have ethnicity data for each participant. However, country level data are nonetheless appropriate given the aims and scope of the investigation. The MIT Committee on the use of Humans as Experimental Subjects (COUHES) approved this study protocol.

### Procedure

Each participant watched a set of 10 ads and men and women in each country saw the same ads. The order of the videos in each set was randomized. At the start of the survey participants were asked for permission to stream videos captured from their webcam to an Internet server. The participants provided consent through the online survey platform. Then they were instructed to watch 10 video ads and after the 10th ad they were asked: How **COMFORTABLE** did you feel during the study from “very uncomfortable” to “very comfortable” on a 5-point scale. Eighty-eight percent reported “neutral” to “very comfortable”, 12% reported below “neutral” to “very uncomfortable.” These latter participants were excluded from further analysis as their behavior was assumed to be atypical. Thus, we have recordings of responses to 10 videos from 879 females (47.2%) and 983 males (52.8%), which totaled 18,620 videos from 1,862 participants. The video recordings comprised a total of 5,471,316 frames. We automatically quantified facial actions in each video frame. We estimate it would have taken approximately 2,000 hours of manual coding to code this material. Videos in which a face was not detected in at least 20% of frames were not used, as the facial base rates were deemed less reliable. Based on this criteria 11.8% of videos were excluded.

### Automated facial coding

While the aim of this paper is not to validate the performance of automated facial action coding classifiers, we describe how the classifiers were created as this is important for replication. Due to the challenging nature of detecting spontaneous facial actions from videos collected over the Internet, we created facial action classifiers, training and validating them against other data collected over the Internet. The resulting classifiers are available as part of the AFFDEX software development kit (SDK) [29], which is available publicly to all researchers.

Inner brow raises were defined as contractions of the *frontalis*, *pars medialis*, muscle which pulls the eyebrows together and up (AU 1 in FACS). Outer brow raises were defined as contractions of the *frontalis*, *pars lateralis*, muscle which pulls the outer eyebrow vertically upwards (AU 2 in FACS). Brow furrows were defined as contractions of the *corrugator* muscle, which pulls the eyebrows down and together (AU 4 in FACS). Smiles were defined as contractions of the *zygomatic major* muscle, which pulls the lip corners toward the ears (AU 12 in FACS). We capture both “Duchenne” and “non-Duchenne” smiles. Lip corner depressors were defined as contractions of *depressor anguli oris* muscle, which pulls the lip corners down (AU 15 in FACS).

#### Coding

The computer classifiers need to be trained to discriminate between images with facial actions present and those without. To create a set of training data for the classifiers, 20 trained FACS coders manually coded a separate set of videos (person independent from the videos analyzed in the present study). Three coders coded each frame of the videos for the presence of the actions. Inter-coder reliability, free marginal *κ*, was measured in terms of frame-by-frame agreement which was found to be moderate to very high (*κ* = .74 for smiles, *κ* = .79 for brow furrows, *κ* = .75 for brow raises, *κ* = .68 for lip depressors, *κ* = .54 for inner brow raises). This agreement is especially notable considering that some of the videos were dark and/or contained facial occlusions.

#### Image feature extraction and classification

In order to detect whether an action appeared in each frame of the video or not, image features were extracted on a frame-by-frame basis. The OpenCV face detector [30] was used to identify the largest face within the image. A custom facial feature point tracker, using a supervised descent method (SDM), was applied within this face region to identify 34 landmark points on the subject’s face. This method of face alignment uses shape and appearance information and a mathematical optimization to identify the most likely positions of the landmarks in each video frame. The image region of interest (ROI) was normalized using the outer eye and mouth points by performing a rotation to align the eyes horizontally and scaling to a uniform size. The resulting ROI contained the whole face including eyes, mouth and nose. Histograms of oriented gradient (HOG) features were extracted from the resulting image ROI. These image features capture the number and direction of gradients and edges within the image ROI. Further details of the image processing can be found in Senechal et al. [31].

For this study we implemented support vector machine (SVM) classifiers for detecting the facial actions. We trained the classifiers on the coded images and tested them on a separate set of images in order to ensure valid performance. The free marginal *κ* is presented as it captures the overall agreement well even in the case of unbalanced class sizes [32]. In the testing data there were 1,571 videos, 708 of males and 863 of females. Our system exhibits state-of-the-art performance in action classification, with consistent performance on both men and women. Inter-coder reliability, free marginal *κ*, was measured in terms of frame-by-frame agreement which was found to be very high. Smiles, *κ* = .787 (Δmale:female = .086), brow furrows, *κ* = .825 (Δmale:female = .006), brow raises *κ* = .903 (Δmale:female = -.003), lip depressors *κ* = .954 (Δmale:female = .009), inner brow raises *κ* = .939 (Δmale:female = .069).

### Statistical analysis

We used mixed effects models for modeling the relationships between gender and the frequency and durations of the facial actions. For each face recording we calculated the percentage of the frames in which each action was detected relative to the total number of available frames. This action base rate captures the duration the action was present for an individual during a video. First, we analyzed the differences between the number of videos in which each action was present versus not present using a binomial linear mixed effects model. Second, of those participants that did exhibit a certain action, we analyzed the differences in durations of the action using a linear mixed effects model. In all of the analyses ad and country were added as uncentered effect codes.

#### Presence of actions

There was a gender difference in the percentage of recordings in which actions were seen. We built a generalized linear mixed effects model with a binomial link function with the ad and country treated as random effects.

$$\begin{array}{c} binomial\left(\text{presence}\right)∼B_{0}+B_{1}*Gender+Z_{1}*Ad+Z_{2}*Country+E \end{array}$$ (1)

Here, B_0_ is an intercept, B_1_ is the parameter that estimates the marginal linear effects of Gender on the likelihood of observing the presence of the action, Z_1_ and Z_2_ are parameters describing the variance in the likelihood of observing an action that can be explained by the differences among ads and countries, respectively. E is an error term. Modeling ad and country as random effects, means we do not want to quantify the specific effect of any one ad or country but rather to account for the overall variability they exert on the likelihood of the action. In addition to presenting results across all countries we also present results for each country individually in which case the country random effect is removed.

#### Durations of actions

As the duration data are close to log-normally distributed we transformed it into log-space and then applied a linear mixed effects model. We built the following model with the ad and country treated as random effects:

$$\begin{array}{c} log\left(\text{duration}\right)∼B_{0}+B_{1}*Gender+Z_{1}*Ad+Z_{2}*Country+E \end{array}$$ (2)

Parameters are similar to those described for Eq (1) but related to the log-transformed duration of the actions. Once again we test effects for all countries combined and for each country individually (in which case the Country random effect is removed.).

## Results

### Gender differences in presence of facial actions

**Fig 1:**
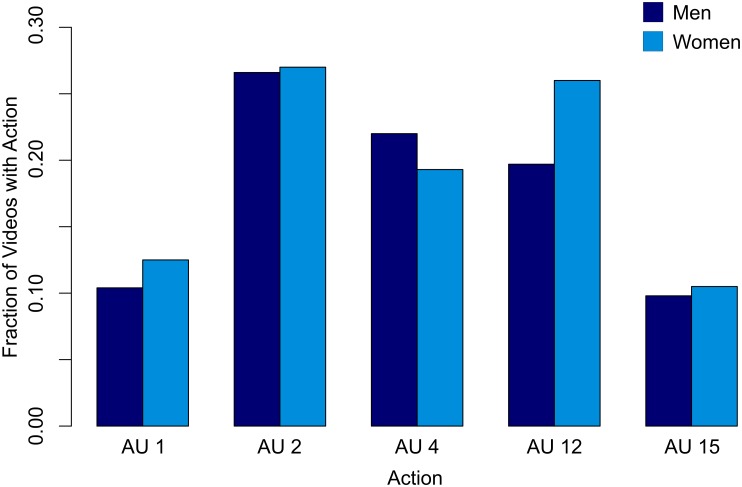
Frequency of facial actions in men and women. The mean fraction of videos in which inner brow raises, outer brow raises, brow furrows, lip corner pulls and lip corner depressors appeared.

Table 1 shows the results for the presence of actions within the videos of males and females and results for the action duration models. Odds greater than one reflect an action that was more frequently observed in females. In addition, we show the valence odds for each of the actions based on a prior large study [25] of facial behavior. Odds greater than one reflect an action that was more frequently associated with positive valence. Fig 1 shows the mean fraction of videos in which inner brow raises, outer brow raises, brow furrows, lip corner pulls and lip corner depressors appeared.

**Table 1:** Regression coefficients for facial action presence and durations (base rates). A frequency odds ratio > 1 means the action was present in responses from more women than men. Lip corner pulls were significantly more frequent and longer in duration in women, inner brow raises were significantly more frequent in women and brow furrows were significantly more frequent and longer in duration in men.

	Valence	Frequency			Duration		
Facial Action	Odds Ratio [25]	Odds Ratio	F-value	*p*-value	Est.	S.E.	*p*-value
AU 1: Inner Brow Raise	0.79**	**1.23**	15.9	< .01	.01	.07	.89
AU 2: Outer Brow Raise	0.66*	1.02	0.06	.806	.00	.04	.99
AU 4: Brow Furrow	0.47***	**0.847**	17.5	< .01	**-0.18**	.05	≪ .01
AU 12: Lip Corner Pull	4.84***	**1.43**	73.4	< .01	**0.14**	.05	≪ .01
AU 15: Lip Corner Depressor	†	1.08	0.89	.345	**-0.15**	.11	.02

* *p* < 0.05,

** *p* < 0.01,

*** *p* ≪ 0.01,

^†^ too few observations in the study.

#### Inner brow raise

Inner brow raises were present in 11.4% of videos with women raising their inner brows in 12.5% of videos and men raising their inner brows in 10.4% of videos. This effect was highly significant (F = 15.9, *p* < 0.001) overall. No significant difference in the duration of inner brow raises was observed.

#### Outer brow raise

Outer brow raises were present in 26.8% of videos. No statistically significant difference was observed in the frequency or duration of brow raises between men and women.

#### Brow furrows

Brow furrows were present in 20.7% of videos, with women furrowing their brows in 19.3% of videos and men furrowing their brows in 22.0% of videos. This effect was highly significant (F = 17.5, *p* < 0.001) overall. Brow furrows from women were significantly shorter in duration than those from men (-0.18, *p* < 0.01).

#### Smiles

Smiles were present in 22.8% of recordings, with women smiling in 26.0% of videos and men smiling in 19.7% of videos. This effect was highly significant (F = 73.4, *p* < 0.01). Smiles from women were significantly longer in duration than those from men (0.14, *p* < 0.01).

#### Lip corner depressor

No statistically significant difference was observed in the frequency of lip corner depressors between men and women. Lip corner depressors from women were significantly shorter in duration than those from men (-0.15, *p* = 0.02).

### Gender differences in facial actions across countries

The results were consistent across different countries with effect sizes differing but not the directionality of any significant results. The smile effect was significant in three (Germany, UK, US) of the five countries. Women smiled in a greater percentage of videos in Germany (Δ = 6.06%), the UK (Δ = 5.65%) and the US (Δ = 8.76%). The brow furrow effect was significant in two (Germany and the UK) of the five countries. Women furrowed their brows in a smaller percentage of videos: Germany (Δ = -4.06%) and the UK (Δ = -7.65%). The inner brow raise effect was significant in two (Germany, China) of the five countries. Women showed more inner brow raises in a greater percentage of videos in Germany (Δ = 3.8%) and China (Δ = 10.5%). Whilst these results are not conclusive as people in different countries were watching different material they provide evidence that the results are robust to cultural differences.

The facial action base rate data are available to researchers (see Supporting Information).

## Discussion

Our first hypothesis, that women will express facial action units more frequently than men was partially supported by the results. Although not all actions may be considered equal the absolute number of detected actions was significantly greater for women compared to men. However, not all actions were expressed more frequently by women. Significantly more smile and inner brow raise actions were observed from women. Significantly more brow furrows were observed from men. For brow raises and lip depressors no significant difference in frequency was observed, although the duration of lip depressors was significantly shorter for women. These results support prior work that women are generally more expressive than men [1, 2, 5, 6]. More frequent smiling amongst women is also expected [7]. However, the results show that this is not universal for all states/actions.

Our second hypothesis, that differences between men and women will be greatest for actions associated with positive valence was partially supported by the data. The largest differences were observed in smiling behavior, with women smiling significantly more. Women also showed brow furrows less frequently. The combination partially supports the idea that women’s facial expressions may be subject to socialization pressures to be positive (greater amounts of smiling and reduced brow furrowing). Across 48 countries, a recent study showed that adults reported that happiness was more desirable for girls than boys [33]. However, inner brow raising also occurred significantly more in women than men. Inner brow raising has been found to be significantly correlated with negative emotional valence [25] and states (fear and sadness) [34]. A norm that requires women to display more positive valence and less negative valence than men may be true on the whole but does not explain gender differences for all actions. It is likely that socialization pressures are specific to discrete emotional states and contexts. For example, socialization pressures on expression of anger are likely to be different to those on expressions of sadness.

Our third hypothesis, that men will express actions associated with states of anger more than women was supported by the data. We observed significantly more brow furrowing in men compared to women. Brow furrowing is associated with prototypic expressions of anger [18]. Although, as people watch advertisements brow furrowing may reasonably be explained by increased cognition or confusion, rather than anger. It is interesting that brow furrowing was significantly reduced in women. Inner brow raises are associated with prototypic expressions of sadness and fear. We observed significantly more inner brow raises from women than men. This finding would support other meta-analyses that found greater expressivity by girls in “internalizing emotions” [35], of which sadness would be one. Our results suggest that there is not a universal norm for women to express less negative emotion than men, but that gender differences are state specific.

One might question whether these results vary across countries. We found the largest effect sizes in smiling frequency in the US and smaller effect sizes in gender differences amongst British participants, findings that are also consistent with prior research [7]. In no cases did the country specific results contradict the overall results. Perhaps differences are related to another cultural variable such as “individualism” [36].

Differences exist in how men and women perceive faces [37]. The difference in frequency by which men and women exhibit facial actions in everyday life may influence how people interpret faces overall. For example a greater propensity to furrow their brows may mean that mens’ faces are more often associated with anger. This may help explain empirical results showing differences in the judgments of anger and happiness in men and women. Prior work argues that the effect is perhaps a combination of sexual dimorphism of the face and gender stereotypes biasing the perception [38]. The automated approach we have presented for analyzing differences in actions may allow us to test on a large-scale whether the magnitude of the difference in the frequency of actions is correlated with the magnitude of difference in the judgment of emotion in neutral faces. This would shed more light on whether differences in judgments are due to gender stereotypes biasing the perception.

Finally, we have demonstrated a research method involving large-scale collection and coding of facial data that has important implications for how observational studies can be performed. It is now possible to replicate and extend existing research in more naturalistic settings and with orders of magnitude more participants.

## Conclusion

There is considerable evidence to suggest that gender differences in expressive behavior are significant. However, the generalizability of much of the extant literature has remained uncertain since the number of participants in experiments (in contrast to archival studies) is relatively small (typically less than 100 people). In addition, the data have generally been collected in laboratory contexts with limited external validity.

We used automated facial expression analysis and an Internet-based data collection method to quantify how much men and women spontaneously display facial actions while watching mundane online content in their own homes. We find that women are not universally more expressive across all facial actions. Nor are they consistently more expressive in positive valence actions and less expressive in negative valence actions.

Overall, women express facial actions more frequently than men, and in particular express more actions of positive valence. However, for negative valence actions expressiveness is dependent on the discrete emotional state. Women expressed actions associated with anger less and actions associated with fear and sadness more than men.

To our knowledge this is the largest study of gender differences in facial behavior based on facial responses collected in naturalistic settings. In addition, to revealing new insights the results provide large-scale evidence for a number of previous findings. This work provides compelling evidence for how crowdsourcing and automated coding of facial responses can contribute to the behavioral sciences.

## Limitations and future directions

Although there is good reason to believe that participants in our experiment watched the videos alone, we do not know for certain whether that was the case, as participants were not asked to report it directly. In addition, the participants were aware that the camera was on during the experiment, which may have created a pseudo-social effect. The implication of both is that we do not know whether the facial displays were in response to the advertisements alone. In addition, although we aimed for as representative a demographic profile as possible, the sample is limited to those who had a webcam. This may limit the generalizations that can be made.

## Supporting information

S1 Data Base rates. The facial action base rate data for each facial video are included.
(CSV)
